# Use of continuous positive airway pressure in drainage of pleural effusion: Educational intervention for evidence-based practice

**DOI:** 10.1016/j.clinsp.2024.100499

**Published:** 2024-09-23

**Authors:** Elinaldo da Conceição dos Santos, Adilson Mendes, Daniela Gonçalves Ohara, Hiago Vinicius Costa Silva, Jhéssica Crhistina Veiga Nascimento, João Paulo Rodrigues Pacheco, William Poncin, Gregory Reychler, Juliana Ribeiro Fonseca Franco de Macedo, Adriana Claudia Lunardi

**Affiliations:** a Master's and Doctoral Programs in Physical Therapy of Universidade Cidade de São Paulo, São Paulo, SP, Brazil; b Department of Biological and Health Sciences, Universidade Federal do Amapá, Macapá, AP, Brazil; c Academic Unit of Health Sciences, Universidade Federal de Jataí, Jataí, GO, Brazil; d Department of Health Sciences. Catholic University of Louvain, Belgium; e Department of Physical Therapy of School of Medicine of Universidade de São Paulo, São Paulo, SP, Brazil

**Keywords:** Implementation science, CPAP ventilation, Pleural effusion, Chest drainage

## Abstract

**Objective:**

To create an educational intervention for health professionals and test its effectiveness in implementing the use of CPAP in hospitalized patients with pleural effusion undergoing thoracic drainage.

**Methods:**

This implementation study was developed in 5 hospitals in Brazil and one in Belgium within four phases: (I) Situational diagnosis (professionals and patients’ knowledge about CPAP usage for drained pleural effusion and checking medical records for the last 6 months); (II) Education and training of professionals; (III) New situational diagnosis (equal to phase I); (IV) Follow-up for two years.

**Results:**

65 professionals, 117 patients’ medical records, and 64 patients were enrolled in this study. Initially, only 72% of medical records presented a description of interventions. CPAP usage was mentioned in only one patient with a chest tube. After phase III, the number of professionals who used CPAP for their patients with drained pleural effusion increased from 28.8% to 66.7%, p < 0.001. Similarly, the acceptability of this therapy for this clinical situation also increased among professionals from 6.4 ± 1.3 to 7.8 ± 1.4, p < 0.001. However, before the implementation, only one medical record described the use of CPAP in one patient with drained pleural effusion. After two years, the use of CPAP therapy by healthcare professionals for patients with drained thoracic drainage was sustained in 3 hospitals.

**Conclusions:**

The educational intervention for the use of CPAP in patients with drained pleural effusion was effective for health professionals. Results were sustained after two years in three of the six hospitals.

## Highlights

• Educational interventions to update healthcare professionals on the importance of CPAP.
• Continuing education needs to be implemented for better patient care.
• An educational intervention may have some success in educating and updating healthcare professionals on the use of CPAP in patients with chest tubes due to pleural effusion.
• Physical therapists need to be strongly encouraged to write down the details of the interventions they perform in patients' medical records.
• New strategies for continuing education of health professionals need to be developed so that the patient receives the best possible care.

## Introduction

Pleural effusion is defined as the abnormal accumulation of fluid in the intrapleural space.^1^ Its origin has different etiologies, including pneumonia, cancer, trauma, heart failure and kidney failure. This abnormal accumulation of fluid in the intrapleural space makes it difficult to slide between the pleurae and can compress the lungs, restricting lung expansion. The main symptoms of pleural effusion are shortness of breath, chest pain, and coughing.^1^

The pleural effusion is a common clinical condition and is associated with complications such as empyema, sepsis, and death.^1^^,^^2^ In the United Kingdom, around 40% of patients with infectious pleural effusion require thoracic surgery and 20% of them will progress to death.^2^ In the United States of America, people out of a thousand develop pleural effusion.^3^ In Brazil, approximately 6.1% of patients with community-acquired pneumonia develop pleural effusion, which appears to be predictive of 30-day mortality.^4^

In general, the prognosis of pleural effusion is favorable in patients diagnosed early and with correct management. To reduce the rate of complications resulting from pleural effusion, chest drainage seems to be the best therapeutic strategy.^5^ However, chest drainage can also lead to complications such as hemothorax or pneumothorax, small and large gauge tube injury or empyema, or bad position of the large-gauge tube in the pleural cavity.^6^ These complications can occur from the time the tube is placed,^5^ during dwelling time, or at the removal of the catheter. In order to reduce such complications and to promote lung aeration, the use of Continuous Positive Airway Pressure (CPAP) in patients with pleural effusion and chest tubes has been proposed.^7^, ^8^, ^9^

Using CPAP with higher pressure generates increased intrathoracic pressure, facilitating the drainage of pleural fluid through the drain. This accelerates the recovery of respiratory function, allowing earlier removal of the chest tube and shortening the length of hospital stay as reported by Santos and colleagues in a randomized controlled trial with 151 patients with drained effusion pleural underwent CPAP with 15 cm H_2_O during consecutive days.^9^

The knowledge about the effectiveness of using CPAP in this population was developed in a controlled environment, suitable for scientific studies. However, this intervention may not be absorbed as quickly in clinical practice due to a lack of information from professionals and/or a lack of validity of its effects in real life. The delay in the implementation of scientific evidence is a growing concern. Barriers^10^ to translating scientific evidence into clinical practice include the poor ability of researchers to communicate with healthcare professionals, the lack of human and technological resources to reproduce the investigated interventions in real life, and the lack of a mechanism set for the delivery of research results to society.

As a strategy to overcome one of the barriers to implementing the use of CPAP as a complementary treatment for pleural drainage, the effectiveness of the active and direct transfer of knowledge from the researcher to the health professional can be investigated.^11^ Therefore, the aim of this study was to create an educational intervention for health professionals and test its effectiveness in implementing the use of CPAP in hospitalized patients with pleural effusion undergoing thoracic drainage.

## Material and methods

### Study design

An implementation study with pre-post assessment, guided by the Standards for Reporting Implementation Studies (StaRI) statement.^12^

### Study setting and participants

**Fig. 1:**
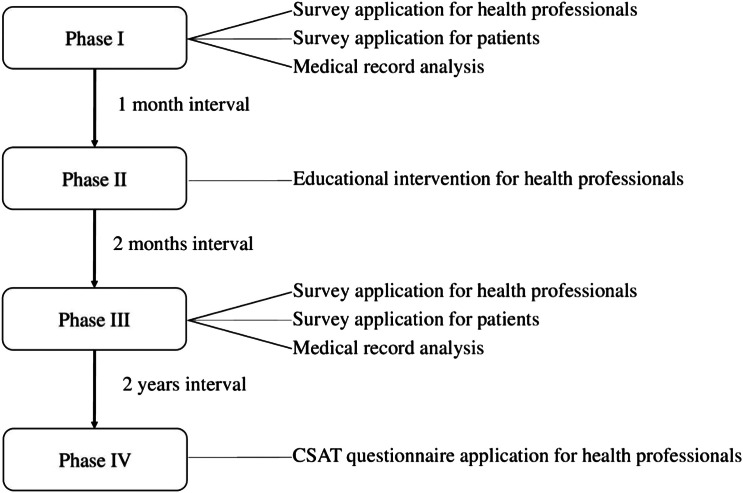
Study phases: Phase I, Baseline audit phase; Phase II, Implementation phase; Phase III, Post-intervention audit; Phase IV, Follow-up. CSAT, Clinical Sustainability Assessment Tool.

Health professionals and patients from five hospitals in Brazil and one in Belgium were enrolled in the study, from March 2019 to January 2023. The authors invited health professionals who work in hospitals where patients with pleural effusion are treated, including physiotherapists, doctors, nutritionists, psychologists and nurses. This study received approval from the Research Ethics Committee of the Universidade Federal do Amapá (protocol: 95602418.5.0000.0003), and from the *Comité d’Éthique Hospitalo-Facultaire* (2019/28AOU/373, protocol number C-PANCH-001). Informed written consent from all participants was obtained. This study is registered on ClinicalTrials.gov (NCT03896672). This implementation study was developed in four phases: (I) Situational diagnosis (by assessing the knowledge of professionals and patients about CPAP usage in the treatment of drained pleural effusion and checking medical records for the last 6 months); (II) Education and training of professionals (using lectures, discussions, and printed material); (III) New situational diagnosis (reassessment of professionals and patients knowledge and recheck of medical records in the last 6 months); (IV) Follow-up for two years (Fig. 1). Detailed description of these phases are provided below.

### Eligibility criteria for health professionals

Working in sectors that directly assist patients with drained pleural effusion in the hospitals involved in this study. Health professionals who failed to participate in phases II and/or III of this study after having participated in phase I were excluded.

### Eligibility criteria for patients

Aged 18 years or older, with pleural effusion diagnosed by the physician and submitted to chest drainage for more than 24 hours, at the time when phase I or phase III was being conducted in that hospital. Patients with contraindications for the use of CPAP, such as drowsiness, restlessness, treatment refusal, hemodynamic instability, shock (systolic blood pressure < 90 mmHg), facial trauma, ineffective cough or swallowing impairment, vomiting, upper gastrointestinal bleeding, acute myocardial infarction in the past 48 hours, or bullous emphysema were excluded.^13^

The health professionals who participated in phases I and III were the same, whereas patients who participated in phases I and III were different individuals.

### Phase I

This phase was carried out in three approaches to make a situational diagnosis about the clinical practice of the use of CPAP in patients with drained pleural effusion. (i) Distribution of a survey to health professionals who managed patients with thoracic drainage, (ii) Distribution of a survey to patients with thoracic drainage due to pleural effusion, and (iii) Analysis of medical records of patients with thoracic drainage due to pleural effusion (6-month retrospective analysis). In each hospital, an audit team was created and composed of two local members selected from researchers, clinical director, administrative director, technical director, coordinator, or head of physiotherapy/rehabilitation unit-department to identify potential barriers to the implementation process and to propose solutions to overcome these barriers. As a strategy to increase health professionals' participation, the audit team of each hospital was responsible for the intervention (Phase II). Audit teams identified barriers to be addressed and helped the research team to develop strategies to address these barriers.

### Phase II

This phase was performed one month after Phase I. In this phase, the results extracted from Phase I as well as the best available evidence on the use of CPAP in patients with drained pleural effusion were presented to health professionals who participated in Phase I.^9^^,^^14^, ^15^, ^16^ All professionals received the following educational strategies: lectures (the same in each hospital) with posters, stickers, and buttons in the conference room; electronic messages (via WhatsApp) with the files of the scientific studies used during the lectures and reminders about the evidence of the use of CPAP in people with drained pleural effusion. Furthermore, the study team was available to clarify and reinforce theoretical and technical content related to the management of CPAP equipment, in case any doubts arose for any of the professionals during phases II and III of the study. For health professionals who were unable to participate in the lectures, the scientific evidence was presented in person, at a time and place that was safe for each participant. All approaches were carried out in the most accessible way possible in all hospital environments to minimize vision.

Aiming to increase the chances of using CPAP, a full kit (1 injector, 1 vaporizer, 1 “T” connection, 1 manual valve, 1 oxygen pressure regulator, 2 face masks for inhalation with inflatable cushion, and 1 head attachment gear for the mask) was donated to each enrolled hospital whenever the equipment was not available onsite. In addition, clinical training to use the equipment was offered to health professionals.

### Phase III

This phase was performed 2 months after Phase II and involved the same health professionals who participated in the previous phases. This phase was designed similarly to phase I, i.e., the same three approaches were used to assess whether there was an increase in CPAP use in patients with drained pleural effusion and to make a new situational diagnosis.

The new survey with the health professionals occurred only once in phases I and III, using a structured form. This survey took place in the hospital, office, home or other place and time at the physical therapist's convenience.

The survey with the patients (who have already received or were receiving physical therapy, in order to check what has been done in their physiotherapeutic treatment) was held by means of a structured form.

### Phase IV

This phase was performed two years after phase III. In this phase, the healthcare professionals involved in the previous phases were surveyed to assess the maintenance of the proposed intervention (phase II) in the real condition of each hospital. This aspect was called the sustainability of implementation.

### Outcome measurement

All the following outcomes were evaluated in Phase I and Phase III, except for sustainability which was only evaluated in Phase IV.

#### Fidelity

The degree of implantation of the use of CPAP in the treatment of drained pleural effusion was evaluated by the adherence of the health professionals and patients. Adherence was assessed using a questionnaire.^17^^,^^18^ The question asked to the health professionals to check fidelity was “Do you use CPAP in your patients with drained pleural effusion?” and the response options were “yes” and “no”. The data was analyzed using the percentage of responses: yes.

#### Reach

The intention to use CPAP in the treatment of drained pleural effusion was assessed using a questionnaire applied to the health professionals.^19^ The question asked to the health professionals to check reach was “Currently, do you intend to use CPAP to treat your patients with drained pleural effusion*?*” and the response options were “yes” and “no”. The data was analyzed using the percentage of responses: yes. If the health professional answers “yes”, the “intention to use CPAP” was measured on a Visual Analog Scale (VAS) with a score from 0 (no intention) to 10 (total intention).

#### Appropriateness

Was evaluated through the health professional's perception of the appropriateness of using CPAP for the treatment of patients in the hospital environment. This outcome was assessed using a questionnaire applied to the health professionals.^20^ The question asked to the health professionals to check appropriateness was “Currently, considering the reality of the hospital environment and your technical ability, do you think it is appropriate to use CPAP to treat your patients with drained pleural effusion?” and the response options were “yes” and “no”. The data was analyzed using the percentage of responses: yes. If the health professional answers “yes”, the “adequacy of CPAP use” was measured on a VAS ranging from 0 (totally inadequate) to 10 (totally adequate).

#### Acceptability

Is the perception of health professionals whether the treatment with CPAP is acceptable?^20^ In this study, acceptability was assessed by health professionals’ level of satisfaction using CPAP. A VAS ranging from 0 (totally unsatisfied) to 10 (totally satisfied) was used.

#### Feasibility

The extent to which the treatment with CPAP can be successfully used within the setting and working conditions of the health professional.^21^ It was evaluated through the success or unsuccess of the use of CPAP in the treatment of drained pleural effusion within the hospital environment in patients’ medical records. If medical records reported the use of CPAP for such clinical situations, feasibility success was determined if there was a decrease in (i) Pulmonary complications, (ii) Need for antibiotics, and (iii) Dwelling time of chest tube, (iv) Length of hospital Stays (LoS). The pulmonary complications considered were pneumonia and atelectasis with clinical repercussions (need for oxygen supplementation). They were recorded from the medical record, laboratory tests, and imaging tests (X-Ray and computed tomography).

#### Tolerance

Was assessed in patients using the visual analog scale ranging from zero to ten for the CPAP use. Zero equals fully tolerable (no discomfort) and ten equals totally intolerable (unbearable discomfort).

#### Sustainability

Is the permanence of health professionals’ understanding of the benefits of CPAP for a long period after the educational intervention.^22^ In this study, sustainability was assessed 2 years after phase III.^23^ The Clinical Sustainability Assessment Tool (CSAT) was used.^24^ The CSAT is a self-administered questionnaire for self-assessment and was answered by the physiotherapy service coordinator only in hospitals that showed a statistical difference in any of the outcomes evaluated between phases I and III. The CSAT assesses 7 domains (Each domain has 5 questions): “engaged and leadership”, “engaged stakeholders”, “organizational readiness”, “workflow integration”, “implementation and training”, “monitoring and evaluation” and “outcomes and effectiveness”. Each question can be scored from 1 (program has this to no extent) to 7 (program has to the full extent) or NA (not able to answer). In each domain, the arithmetic mean between the questions is obtained (score from 1 to 7). Then, the arithmetic mean between the domains is obtained (score from 1 to 7). Thus, reporting the final CSAT sustainability score. The domains with lower average scores indicate areas where your practice's capacity for sustainability could be improved.^24^

### Statistical analysis

The sample of health professionals was based on intentional sampling. In addition, a sample of 64 (32 in Phase I and 32 in Phase III) patients was calculated considering a small effect size^25^ of the educational intervention of 30%, with 80% power and 5% alpha, using the paired *t*-test in GPower 3.1 software. The *t*-test was used to compare independent samples for continuous variables and the Mann-Whitney test for dichotomous variables, using the BioEstat 5.3 software.^26^

## Results

Although other professionals who make up the healthcare team were invited to participate in the study, only physiotherapists and doctors accepted the invitation. In total, coming from the 5 hospitals involved in this study, 60 physiotherapists and 5 doctors were included. Twenty-one out of 65 (32%) included health professionals have the degree of specialist in intensive or respiratory care. Sixty-four patients (32 in Phase I and 32 in Phase III) were included. In addition, 117 medical records (87 in Phase I and 30 in Phase III) were analyzed. Initially, in 72% of medical records which presented a description of interventions, CPAP usage was mentioned in only one patient with a chest tube. Baseline characteristics of healthcare professionals, patients, and medical records for each hospital are shown in Table 1.

**Table 1:** Included groups of population baseline characteristics.

Hospitals		Characteristics of health professionals				Characteristics of patients								Medical records analyzed	
Country	Beds	n	Male, n (%)	Age (years)	Time since graduation, n (%)^a^	n	Male, n (%)			Age (years)		Cause of drainage, n (%)^a^			n
Brazil						Phase I	Phase III	Phase I	Phase III	Phase I	Phase III	Phase I	Phase III	Phase I	Phase III
A	113	20	11 (55)	38 ± 9.6	11 to 20 yrs, 8 (40)	16	13	12 (75)	13 (100.0)	28.1 ± 8.2	37.9 ± 19.6	Trauma, 14 (87.5)	Trauma, 13 (100)	50	11
B	132	8	3 (37.5)	35.1 ± 6.5	11 to 20 yrs, 4 (50)	5	9	5 (100)	7 (77.8)	30.4 ± 9.6	32.2 ± 7.5	Trauma, 3 (60)	Trauma, 8 (88.9)	18	11
C	181	21	5 (23.8)	38 ± 8.6	11 to 20 yrs, 9 (42.8)	2	2	2 (100)	2 (100.0)	18.5 ± 0.71	40 ± 29.7	Cancer, 2 (100)	Cancer, 2 (100)	2	0
D	70	5	2 (40)	29 ± 1	< 5 yrs, 3 (60)	5	4	4 (80)	4 (100.0)	33.2 ± 16.3	31.5 ± 9.5	Trauma, 3 (60)	Trauma, 4 (100)	13	4
**Belgium**															
E	979	11	4 (36.4)	38.4 ± 12.5	11 to 20 yrs, 4 (36.4)	4	4	2 (50)	2 (50.0)	65.8 ± 12.0	64.7 ± 5.4	Cancer, 2 (50)	Cancer, 4 (100)	4	4
Total	1,475	65	25 (38.5)	37.7 ± 9.4	11 to 20 yrs, 25 (37.9)	32	32	25 (78.1)	28 (87.5)	33.3 ± 16.1	39 ± 17.6	Trauma, 20 (62.5)	Trauma, 25 (78.1)	87	30

n, Absolute number; %, Percentage; SD, Standard Deviation.

a Most chosen or observed option among the participants.

Considering the results of the 5 hospitals that participated in the study together, fidelity, acceptability, and appropriateness increased after the educational intervention compared to Phase I (Table 2). However, only 13% of the medical records had a description of interventions, and a single patient with a chest tube used CPAP. Analyzing each hospital individually, fidelity improved in three hospitals, acceptability improved in two hospitals, and appropriateness improved only in one of the five included hospitals (Table 2). There was no difference in patients’ tolerance for the use of CPAP between phases I and III (7.2 ± 0.9 vs. 7.2 ± 0.95; p = 1.0). There was no statistical pre-post intervention difference in the selected clinical outcomes in any hospital, either taken individually or together (Table 3).

**Table 2:** Outcomes of the implementation of scientific evidence before and after the health professional's education intervention.

Hospitals (professionals)	Fidelity			Reach			Acceptability (score from 0 to 10)			Appropriateness		
	Phase I	Phase III	p-valor	Phase I	Phase III	p-valor	Phase I	Phase III	p-valor	Phase I	Phase III	p-valor
**Brazil**	**n (%)**	**n (%)**		**n (%)**	**n (%)**		**Mean ± SD**	**Mean ± SD**		**n (%)**	**n (%)**	
A (n = 20)	4 (20)	15 (75)	**0.01**	16 (80)	20 (100)	0.14	5.75 ± 0.96	8.5 ± 0.74	**<0.0001**	15 (75)	18 (90)	0.21
B (n = 8)	5 (62.5)	7 (87.5)	0.20	8 (100)	8 (100)	1.00	6.2 ± 1.3	8.1 ± 1.57	**0.02**	8 (100)	6 (75)	0.20
C (n = 21)	8 (30.1)	15 (71.4)	**0.03**	19 (90.5)	18 (85.7)	0.40	7.1 ± 1.3	7.6 ± 1.4	0.19	14 (66.7)	20 (95.2)	**0.01**
D (n = 5)	0 (0.0)	4 (80)	**0.02**	5 (100)	5 (100)	1.00	0.0 ± 0.0	7.25 ± 0.95	**<0.0001**	3 (60)	5 (100)	0.15
**Belgium**												
E (n = 11)	2 (18.1)	3 (27.2)	0.36	6 (54.5)	7 (63.6)	0.36	5.5 ± 0.7	5.3 ± 0.6	0.39	6 (54.5)	9 (81.8)	0.14
Total (n = 65)	19 (28.8)	44 (66.7)	**<0.0001**	55 (83.3)	59 (89.4)	0.27	6.4 ± 1.3	7.8 ± 1.38	**0.0002**	47 (71.2)	59 (89.4)	**0.03**

n, Absolute number; %, Percentage; SD, Standard Deviation.

**Table 3:** Outcomes of the implementation of scientific evidence checked from patients’ medical recorded before and after the health professional's education intervention.

Hospitals (patients’ medical record in phase I; phase III)	Feasibility											
	Pulmonary Complications, n (%)			Need for antibiotics, n (%)			Dwelling time of chest tube (in days, mean ± SD)			LoS (in days, mean ± SD)		
	Phase I	Phase III	p-valor	Phase I	Phase III	p-valor	Phase I	Phase III	p-valor	Phase I	Phase III	p-valor
**Brazil**												
A (n = 50; 11)	16 (32.0)	1 (9.1)	0.16	39 (78.0)	5 (45.5)	0.06	5.5 ± 3.3	3.9 ± 1.9	0.06	6.1 ± 3.4	4.2 ± 2.2	0.08
B (n = 18; 11)	7 (38.8)	1 (9.1)	0.11	16 (88.9)	8 (72.7)	0.34	5.8 ± 4.9	5.5 ± 6.1	0.89	9.4 ± 15.4	6.4 ± 6.8	0.55
C (n = 2; 0)	0 (0.0)	–	–	2 (100)	–	–	3.0 ± 0.0	–	–	3.5 ± 0.7	–	–
D (n = 13; 4)	4 (30.7)	1 (25.0)	1.00	11 (84.6)	2 (50.0)	0.22	5.6 ± 1.6	2.75 ± 0.9	**0.04**	9.5 ± 9.4	3.0 ± 0.8	0.20
**Belgium**												
E (n = 4; 4)	2 (50.0)	1 (25.0)	1.00	4 (100)	3 (75.0)	1.00	12.2 ± 1.2	10 ± 4.6	0.39	14.7 ± 2.3	12.5 ± 4.0	0.38
**Total** (n = 87; 30)	29 (33.3)	4 (13.3)	0.06	72 (82.7)	18 (60.0)	**0.02**	5.8 ± 3.7	5.1 ± 4.6	0.40	7.6 ± 8.4	6.0 ± 5.2	0.33

n, Absolute number; %, percentage; SD, Standard Deviation; LoS, Lenght of Hospital Stays.

Although patients' medical records show no real increase in CPAP use in clinical practice, the sustainability of knowledge that health professionals acquired during the educational phase was detected in 3 out of 5 hospitals. In a range from one to seven points, the total scores in those hospitals were 6.1 ± 0.5, 6.0 ± 0.8, and 5.4 ± 1.7, respectively. The scores for each item were Engaged Staff and Leadership = 6.4/5.6/5.4, Engaged Stakeholders = 5.4/5.4/6.6, Organizational Readiness = 5.4/6.2/5.6, Workflow Integration = 6.8/7.0/6.8, Implementation and Training = 6.0/6.2/5.0, Monitoring and Evaluation = 6.0/4.6/1.8, and Outcomes and Effectiveness = 6.6/7.0/6.8.

## Discussion

The results of this study show that an educational intervention for the use of CPAP in patients with drained pleural effusion was effective in improving the knowledge of health professionals in half of the included hospitals, with maintenance of this newly acquired knowledge after two years. However, this knowledge did not translate into change in clinical practice. In addition, this study showed that there is a clear underreporting of notes of the interventions in the medical reports.

Before the intervention, the fidelity analysis showed that most healthcare professionals surveyed in this study thought that the CPAP technique was not adequate to be used for patients with pleural effusion and chest drainage. The reasons for these results could be the lack of material resources and the lack of knowledge about how to apply CPAP. After Phase II, the fidelity and acceptability increased in three out of six hospitals. It is probable that the support via the CPAP kit provided to the hospital and the training of professionals in the use of this material improved the comfort in using this therapy. However, there are contradictory reports between what physical therapists think and the implementation of CPAP therapy in patients with drained pleural effusion into clinical practice. Nevertheless, as physical therapists did not report their treatments it in medical records, it is difficult to detect the effectiveness of the educational intervention to implement CPAP therapy in this clinical situation.

On the other hand, the implementation of the use of CPAP failed in three hospitals after the educational intervention. This could be explained by some negative reports pointed out in those hospitals such as “lack of human resources”, “incapacity of hospital infrastructure”, “cultural issues”, and “lack of more effective continuing education on the use of CPAP after the end of phase III”. Recently, a study on the implementation of good health practices that also took place in three phases pointed out the importance of educational interventions and adequate infrastructure to increase adherence to practices based on scientific evidence.^27^ In the present study, the material infrastructure, staff and working conditions were not directly evaluated, which does not allow more definitive conclusions to be drawn about the reasons for implementation failure in 3 of the 6 hospitals included. Strategies to overcome these specific barriers have been discussed with appropriate solutions including facilitating the implementation of scientific evidence in the hospital environment.^28^, ^29^, ^30^

The patient's tolerance of CPAP use by patients was high and did not change between phases of this study. In any case, this is information that always needs to be investigated in CPAP sessions because a prospective study with 20 patients who used non-invasive ventilation to treat acute respiratory failure in an Intensive Care Unit, reported that when support and continuous monitoring are offered, mask adherence increases with consequently increased patient's tolerance.^31^

Sustaining the implemented evidence (use of CPAP in the patient with drained pleural effusion) in clinical practice reflects the overcoming of the barriers faced, even after two years. However, despite that the use of CPAP was satisfactorily sustained in three hospitals, in one of them there is a need for improvement in the Monitoring and Evaluation domain to further consolidate the implementation of CPAP usage. Still, considering that the handling of sustainability in implementation research is one of the problems to be considered, due to its complexity that goes beyond the financial factor, e.g. political, structural and epidemiological factors,^32^^,^^33^ in general the authors can interpret this implementation as a success, since changing routines, cultures, beliefs within a health institution is a great challenge.

### Strengths and limitation

To our knowledge, this is the first multicenter study in the field of physiotherapy testing the implementation of scientific evidence using educational intervention and auditing in hospitals. Despite the growing amount of evidence on the effectiveness of interventions performed by physical therapists, studies of knowledge transfer and implementation in clinical practice are still scarce.^34^ The use of auditing is also a strength, ensuring clarity of results in all phases of the study.

Some limitations of this study were detected. The most serious was probably related to the lack of guidance, in the educational intervention, on the importance of reporting the use of CPAP in patient records. The authors anticipated that this would be a common practice for physiotherapists, but the reality detected in the audits was completely different. Additionally, even though other health team professionals were invited to participate in this study, only physiotherapists and doctors accepted the invitation, probably because they are the professionals most directly involved in patients' respiratory care, at least in the culture of the hospitals where the study was carried out. Finally, although some of the clinical outcomes evaluated showed a pre-post intervention statistical difference, it is not possible to confirm that the clinical benefits are real, as the use of CPAP therapy was not reported in the medical records.

## Conclusions

The present results show that an educational intervention may have some success in educating and updating healthcare professionals on the use of CPAP in patients with chest tubes due to pleural effusion. However, this may not be converted into implementation in clinical practice. In addition, physical therapists need to be strongly encouraged to write down the details of the interventions they perform in patients' medical records. New strategies for continuing education of health professionals need to be developed so that the patient receives the best possible care.

## CRediT authorship contribution statement

**Elinaldo da Conceição dos Santos:** Conceptualization, Data curation, Formal analysis, Visualization, Writing – original draft, Writing – review & editing. **Adilson Mendes:** Formal analysis, Visualization, Writing – original draft, Writing – review & editing. **Daniela Gonçalves Ohara:** Formal analysis, Visualization, Writing – original draft, Writing – review & editing. **Hiago Vinicius Costa Silva:** Data curation, Visualization, Writing – review & editing. **Jhéssica Crhistina Veiga Nascimento:** Data curation, Visualization, Writing – review & editing. **João Paulo Rodrigues Pacheco:** Data curation, Visualization, Writing – review & editing. **William Poncin:** Formal analysis, Methodology, Visualization, Writing – original draft, Writing – review & editing. **Gregory Reychler:** Formal analysis, Methodology, Visualization, Writing – original draft, Writing – review & editing. **Juliana Ribeiro Fonseca Franco de Macedo:** Formal analysis, Methodology, Visualization, Writing – original draft, Writing – review & editing. **Adriana Claudia Lunardi:** Conceptualization, Methodology, Project administration, Supervision, Visualization, Writing – original draft, Writing – review & editing.

## Declaration of competing interest

The authors declare no conflicts of interest.
